# Dynamics of Fermentation Parameters and Bacterial Community in High-Moisture Alfalfa Silage with or without Lactic Acid Bacteria

**DOI:** 10.3390/microorganisms9061225

**Published:** 2021-06-04

**Authors:** Shanshan Zhao, Fengyuan Yang, Yuan Wang, Xiaomiao Fan, Changsong Feng, Yanping Wang

**Affiliations:** 1 Henan Key Laboratory of Ion Beam Bio-Engineering, College of Physics, Zhengzhou University, Zhengzhou 450000, China; zsszd@gs.zzu.edu.cn (S.Z.); yangfy@gs.zzu.edu.cn (F.Y.); wangyuany5@163.com (Y.W.); fanxiaomiao1125@163.com (X.F.); 2 Henan Key Laboratory of Ion Beam Bio-Engineering, School of Agricultural Science, Zhengzhou University, Zhengzhou 450000, China; 3 Institute of Animal Husbandry and Veterinary Science, Henan Academy of Agricultural Sciences, Zhengzhou 450000, China; Fengchangsong72@163.com

**Keywords:** alfalfa silage, bacterial community, fermentation quality, high moisture content, high-throughput sequencing, lactic acid bacteria

## Abstract

The aim of this study was to gain deeper insights into the dynamics of fermentation parameters and the bacterial community during the ensiling of high-moisture alfalfa. A commercial lactic acid bacteria (YX) inoculant was used as an additive. After 15 and 30 days of ensiling, the control silage (CK) exhibited a high pH and a high concentration of ammoniacal nitrogen (NH_3_-N); *Enterobacter* and *Hafnia-Obesumbacterium* were the dominant genera. At 60 d, the pH value and the concentration of NH_3_-N in CK silage increased compared with 15 and 30 d, propionic acid and butyric acid (BA) were detected, and *Garciella* had the highest abundance in the bacterial community. Compared with CK silage, inoculation of YX significantly promoted lactic acid and acetic acid accumulation and reduced pH and BA formation, did not significantly reduce the concentration of NH_3_-N except at 60 d, and significantly promoted the abundance of *Lactobacillus* and decreased the abundance of *Garciella* and *Anaerosporobacter*, but did not significantly inhibit the growth of *Enterobacter* and *Hafnia-Obesumbacterium*. In conclusion, high-moisture alfalfa naturally ensiled is prone to rot. Adding YX can delay the process of silage spoilage by inhibiting the growth of undesirable microorganisms to a certain extent.

## 1. Introduction

Alfalfa is a perennial herbaceous legume. It is one of the most important legume forages in the world because of its good nutritional quality, high yield, and strong adaptability [1]. As a source of livestock protein, it can be the basic component in the rations of dairy cattle, beef cattle, horses, sheep, goats, and other livestock [2]. Ensiling has been regarded as a common way for preserving green forages. It is an anaerobic microbial-based fermentation process, dominated by lactic acid bacteria (LAB), which produce the lactic acid (LA) required for pH decline and inhibition of non-acid-resistant harmful microorganisms [3]. In contrast to corn and other cereal crops, alfalfa is considered a difficult silage crop, mainly because of its high buffer capacity and low concentration of water-soluble carbohydrate (WSC) [4]. When ensiled at moisture concentration > 700 g/kg dry matter (DM), it can be a challenge to obtain good-quality silage as this dilutes the WSC concentration and LAB count, and counteracts a rapid drop in pH [5,6]. Moreover, high-moisture silage often bears a high risk of effluent loss and clostridial fermentation, leading to high DM loss, extensive proteolysis, and high butyric acid (BA) production, which can reduce feed palatability [7,8]. Feeding silage of high BA content will reduce animal DM intake and clostridial endospores can even lead to clostridial contamination in milk [9]. In practice, field wilting is a traditional method to reduce water content. In this case, an additional loss may occur due to crushing loss and respiratory consumption. Moreover, the process depends entirely on weather conditions, which can be adverse if rainfall occurs during this period [10]. For example, in the main rain season (from late spring to summer) in East China, alfalfa with suitable water content is not easy to obtain [11]. When the lower WSC content and higher moisture content of raw materials introduce difficulties for natural ensiling, adding additives is one of the most commonly used methods to improve silage quality. At present, there are many kinds of silage additives used in the world, which can be divided into four categories: bacterial inoculants, chemicals, enzymes, and non-protein nitrogen [12]. Each of these additives has its own advantages and limitations. Adding organic acid directly is effective to improve silage quality, and some studies have been reported [13,14]. However, the higher initial cost compared with that of inoculants may be a barrier to adoption [12]. In addition, in order to spray organic acid evenly in the actual production process, especially in large farms, the machinery used may cause corrosion and also harm the safety of operators. Due to the generally recognized as safe (GRAS) status, LAB inoculants have become the effective tool to improve the microbial quality of silage by screening varieties with special characteristics [15]. Commercial LAB inoculants are frequently used to improve the ensiling process. Many types of homo-fermentative LAB, *Enterococcus faecium*, and *Pediococcus* spp. have been proposed as effective stimulants to reinforce LA fermentation [16,17]. LAB YX, as a special commercial LAB additive for alfalfa, has a good effect in improving the quality of alfalfa silage, but it is generally used in alfalfa silage with suitable moisture content. There are few reports about ensiling high-moisture alfalfa when adding LAB YX.

It is widely known that the silage process involves a variety of microbial communities and biochemical reactions. The quality of silage depends largely on the microbial community and its dynamic succession and fermentation metabolites [18]. A better understanding of the microbial community in silage is essential for improving silage quality, especially at high moisture content. In recent years, medium-independent methods have been developed for the analysis of microbial communities to avoid the limitations of traditional culture methods [19,20]. In this study, a next-generation sequencing (NGS) technique was used for studying bacterial communities.

The purpose of this study was to explore the dynamics of fermentation parameters and the bacterial community and the correlations among them in high-moisture naturally ensiled alfalfa, and to explore the effect of adding commercial LAB YX on the dynamic of fermentation parameters and the bacterial community. This will deepen understanding of the role of microorganisms in high-moisture alfalfa ensiling and provide more detailed information for alfalfa ensiling in a humid environment.

## 2. Materials and Methods

### 2.1. Forage Harvest and Silage Preparation

Fresh alfalfa was cultivated and harvested after rain at the early bloom stage in Zhengzhou, Henan Province, China (temperate monsoon climate, 34.76° N, 113.65° E, altitude 110.4 m above sea level). The material was wilted for about 24 h to obtain a DM content of 266.03 g/kg fresh matter (FM). The material was then chopped using a crop chopper into sections of approximately 1–2 cm in length. The pH, WSC, and high buffering capacity (BC) of fresh alfalfa were 6.06, 17.56 g/kg DM, and 460 mEq/kg DM, respectively. The epiphytic LAB, coliform bacteria, and aerobic bacteria in the fresh alfalfa were 4.65, 4.83, and 5.28 log10 cfu g^−1^ FM, respectively. To accurately trace the degradation of the organic acids before and after fermentation, laboratory vacuum-packed mini silos have been frequently used for alfalfa silage [21,22,23]. Approximately 500 g for each of three replicates of chopped alfalfa were packed into polyethylene plastic bags (dimensions: 200 mm × 300 mm; Dongda, Zhengzhou, China), vacuumed, and sealed with a vacuum sealer (P-290, Shineye, Dongguan, China). These were individually prepared for each of the following treatments: (a) control (CK), which was treated with no additive as natural silage and (b) LAB (YX), isolated from the Yaxin alfalfa ensiling additive (Yaxin Biotechnology Co., Ltd., Taiwan, China). The application rate of LAB of YX into the fresh forage was 1 × 10^6^ cfu g^−1^ FM, and an equal volume of distilled water was sprayed onto the fresh alfalfa for the control group. The samples were ensiled for 15, 30, and 60 d at room temperature (25 °C).

### 2.2. Analysis of Fermentation Parameters and Chemical Composition

Immediately after the bags were opened, the subsamples (10 g) were blended with 90 mL of sterilized water. The pH was measured with an electrode pH meter (Mettler Toledo Co., Ltd., Greifensee, Switzerland). The ammoniacal nitrogen (NH_3_-N) level was determined using Berthelot colorimetry [24]. The concentrations of organic acids, LA, acetic acid (AA), propionic acid (PA), and BA were measured using a high-performance liquid chromatography system (Waters Inc., MA, USA; column: Carbomix H-NP 10:8% (7.8 × 300 mm × 10 μm), Sepax Technologies, Inc., Santa Clara, CA, USA; detector: UV detector; Waters Inc.; eluent: 0.0254% H_2_SO_4_, 0.6 mL min^−1^; temperature: 55 °C) [25]. The DM weights of the fresh alfalfa and silage materials were determined following oven drying at 65 °C for 48 h. The oven-dried samples were then milled through a 1.0 mm sieve prior to further chemical analyses. The WSC was determined by anthrone colorimetry [26]. The BC was determined using the hydrochloric acid–sodium hydroxide method [27]. The neutral detergent fiber (NDF), acid detergent fiber (ADF), and acid detergent lignin (ADL) concentrations were determined according to Van Soest et al. [28], using a CXC-6 crude fiber analyzer (Zhejiang Top Instrument Co., Ltd., Zhejiang, China). Hemicellulose and cellulose concentrations were estimated indirectly as [NDF−ADF] and [ADF−ADL], respectively.

### 2.3. Bacterial Community Analyses

Subsamples (10 g) of each fresh or silage sample were shaken well with 90 mL of sterile phosphate buffer saline at 180 rpm for 1 h. The solution was filtered through four layers of medical gauze and the filtrates centrifuged at 8000× *g* for 15 min at 4 °C to collect the microbial pellet for DNA extraction [29]. Total DNA was extracted via a Bacterial DNA Kit D3350-02 (Omega Biotek, Norcross, GA, USA). After DNA extraction, DNA concentration was evaluated by 1% agarose gel electrophoresis. The 16S rRNA genes of distinct regions (16S V4) were amplified using the forward primer 515F (GTGCCAGCMGCCGCGGTAA) and reverse primer 806R (GGACTACHVGGGTWTCTAAT). The resulting PCR products were extracted from a 2% agarose gel and further purified using a GeneJET Gel Extraction Kit (Thermo Fisher Scientific Inc., Carlsbad, CA, USA). The amplicon sequencing of 16S rDNA was conducted using Thermo Fisher’s Ion S5^TM^XL (Biomarker Technologies Corporation, Beijing, China). The NGS reads were assembled using FLASH (version 1.2.11) and low-quality reads were removed according to the QIIME quality control process (version 1.9.1). Sequence analyses were performed via Uparse software (v7.0.1001). A 97% similarity cutoff was used to define operational taxonomic units (OTUs).

### 2.4. Statistical Analyses

The data of fermentation parameters were statistically analyzed using the GLM procedure of IBM SPSS version 22.0 (SPSS Inc., Chicago, IL, USA). Statistical differences in parameters among the days were determined in accordance with Duncan’s multiple comparison test, and effects were considered significant when *p* < 0.05. The alpha diversities of samples, the Shannon index, and Chao richness estimator were determined using Mothur (version 1.30.1, http://www.mothur.org/wiki/Classify.seqs accessed on 24 August 2020). Taxonomic classification at different levels was performed using the Ribosome Database Project (version 2.2, http://sourceforge.net/projects/rdpclassifier/ accessed on 24 August 2020) algorithm to classify the representative sequences of each OTU. Linear discriminant analysis effect size (LEfSe) analysis (Galaxy Version 1.0, https://huttenhower.sph.harvard.edu/galaxy/ accessed on 24 August 2020) was conducted to determine the differentially abundant taxonomies among different treatments by coupling one-way analysis of variance with a non-parametric factorial Wilcoxon sum-rank test for statistical significance using Python (version 2.7, https://www.python.org accessed on 24 August 2020). Non-metric multidimensional scaling (NMDS) analysis and a Spearman correlation heatmap based on the Spearman correlation coefficients among the bacterial community and fermentation parameters were produced using R software (version 2.15.3).

The derived variable regions of 16S rRNA gene sequence datasets were submitted to the NCBI under the BioProject accession number PRJNA720220.

## 3. Results

### 3.1. Changes in Fermentation Parameters in Silages during Ensiling with or without LAB

**Figure 1:**
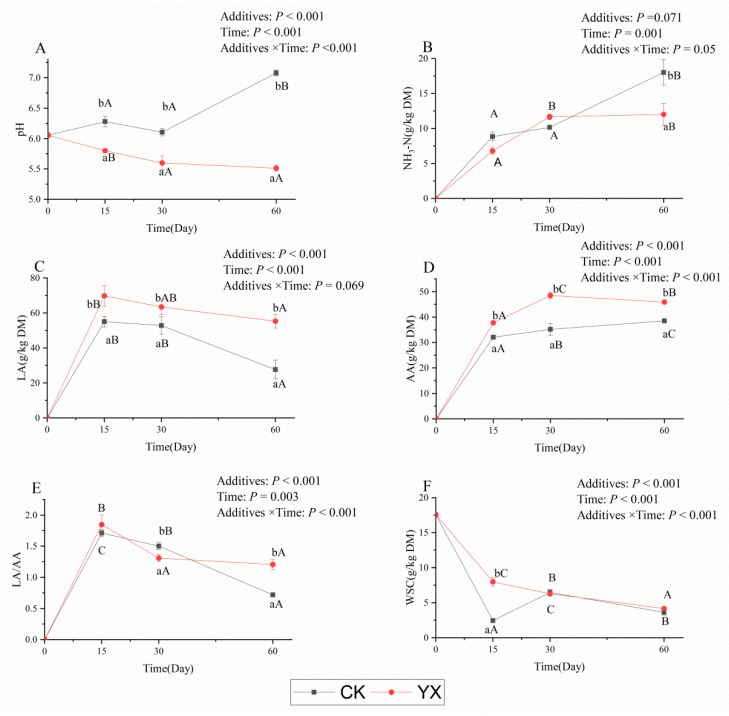
Changes in fermentation parameters ((**A**): pH value; (**B**): NH_3_-N; (**C**): LA; (**D**): AA; (**E**): LA/AA; (**F**): WSC) in alfalfa silages during ensiling for 60 d. Values with different superscript lowercase letters show significant differences among treatments on the same ensiling day, values with different superscript capital letters show significant differences among ensiling days in the same treatment (*p* < 0.05). CK, control; YX, inoculated with commercial LAB YX; NH_3_-N, ammoniacal nitrogen; LA, lactic acid; AA, acetic acid; LA/AA, the ratio of lactic acid to acetic acid; WSC, water-soluble carbohydrate.

Changes in fermentation parameters in silages during ensiling with or without LAB are shown in Figure 1. In the natural silage (CK), neither pH nor NH_3_-N concentration differed between 15 and 30 d, but increased (*p* < 0.05) greatly at 60 d compared with 30 d. The LA concentration and LA/AA peaked at 15 d, then declined, and were significantly (*p* < 0.05) lower at 60 d than at 30 d. The AA concentration significantly (*p* < 0.05) increased with longer ensiling time. The WSC concentration decreased rapidly during 15 d of ensiling, and then remained at 3.62 g/kg DM at 60 d. Both PA and BA were detected at 60 d, with concentrations of 29.73 and 37.13 g/kg DM, respectively.

In the YX silage, the pH and WSC concentration gradually decreased with longer ensiling time; the NH_3_-N concentration increased significantly (*p* < 0.05) from the beginning to 30 d and there was no significant (*p* > 0.05) change from 30 to 60 d. The LA concentration and LA/AA value rapidly increased up to 15 d and then decreased; the AA content significantly (*p* < 0.05) increased up to 30 d and then significantly (*p* < 0.05) decreased by 60 d. No BA was detected in YX silage.

Compared with CK silage, the pH of YX silage was significantly (*p* < 0.05) lower, and the LA and AA concentrations were significantly (*p* < 0.05) higher in all ensiling periods. At 60 d, the NH_3_-N concentration of YX silage was significantly (*p* < 0.05) lower than of CK silage at 60 d. The LA/AA of YX silage was significantly (*p* < 0.05) lower than of CK silage at 30 d, but significantly (*p* < 0.05) higher at 60 d.

**Figure 2:**
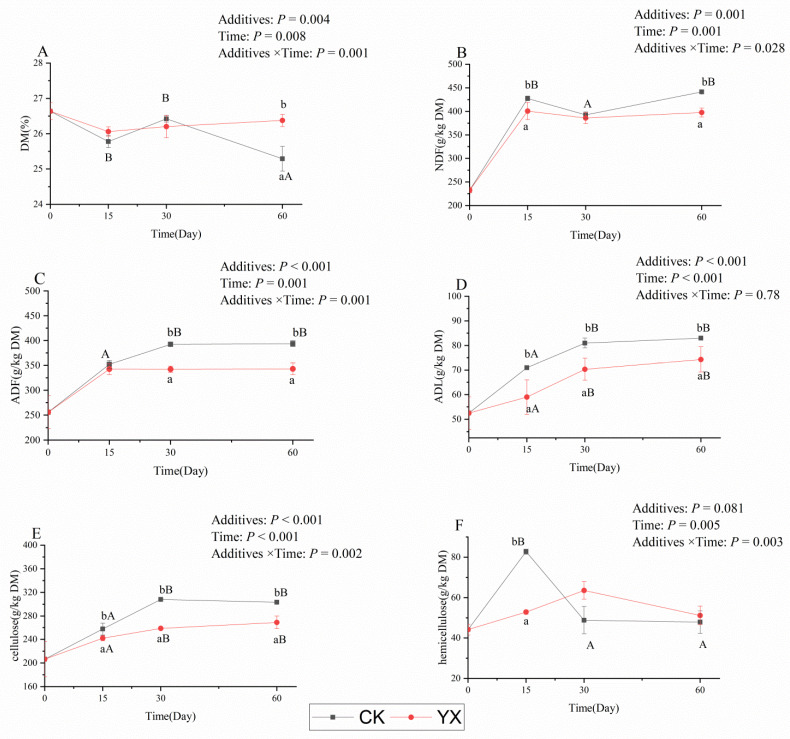
Changes in DM (**A**), NDF (**B**), ADF (**C**), ADL (**D**), cellulose (**E**), and hemicellulose (**F**) in alfalfa silages during ensiling for 60 d. Values with different superscript lowercase letters show significant differences among treatments on the same ensiling day, values with different superscript capital letters show significant differences among ensiling days in the same treatment (*p* < 0.05). CK, control; YX, inoculated with commercial LAB YX; DM, dry matter; NDF, neutral detergent fiber; ADF, acid detergent fiber; ADL, acid detergent lignin.

Changes in DM and structural carbohydrates in silages during ensiling with or without LAB are shown in Figure 2. In the CK silage, the DM content did not change significantly during 30 d of ensiling, but was greatly reduced (*p* < 0.05) at 60 d. Compared with CK silage, the YX silage had significantly higher DM content at 60 d, significantly lower ADL and cellulose content at each ensiling time, significantly lower NDF content at 15 and 60 d, significantly lower ADF content at 30 and 60 d, and significantly lower hemicellulose content at 15 d (*p* < 0.05).

### 3.2. Changes in Bacterial Community in Silages during Ensiling with or without LAB

**Figure 3:**
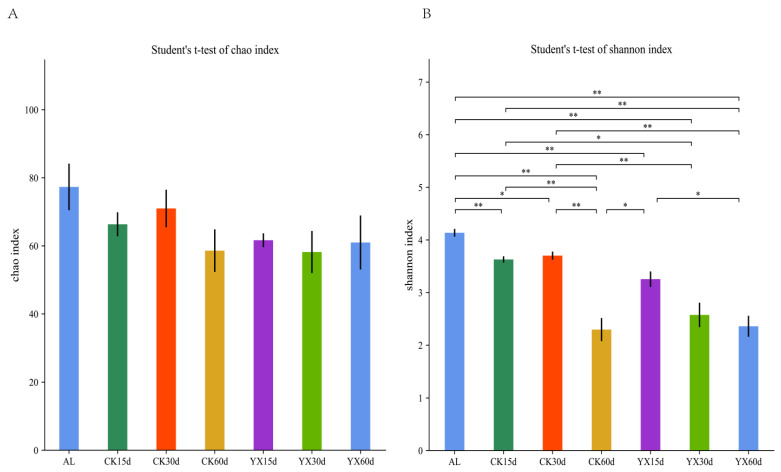
Bar plots of Chao indices (**A**) and Shannon indices (**B**) of bacterial communities in alfalfa silage. AL, alfalfa material before ensiling; CK, control; YX, inoculated with commercial LAB YX; the numbers following CK and YX stand for ensiled days of silage. * *p* < 0.05; ** *p* < 0.01.

High-throughput analyses were used to detect the bacterial diversity of the alfalfa silages at 15, 30, and 60 d. The average number of reads per sample was 84,194, and the average number of effective data was 79,478. The effective rate of quality control was 94.48%. Using 97% identity, the sequences were clustered into 265 OTUs. The Chao and Shannon indexes were used to represent richness and diversity indices of the bacterial communities, respectively. There was no significant (*p* > 0.05) change in the richness of the bacterial community before and after ensiling, and the diversity of the bacterial community significantly (*p* < 0.05) decreased after ensiling (Figure 3A,B). In the CK silage, there was no significant (*p* > 0.05) change in bacterial community richness during ensiling; the diversity of the bacterial community was significantly lower at 60 d compared with 15 and 30 d. The richness of the bacterial community of YX silage was similar to that of CK silage. Compared with CK silage, the bacterial community diversity of YX silage was lower at 30 d (*p* < 0.05), but there was no significant difference between the two at 15 and 60 d.

**Figure 4:**
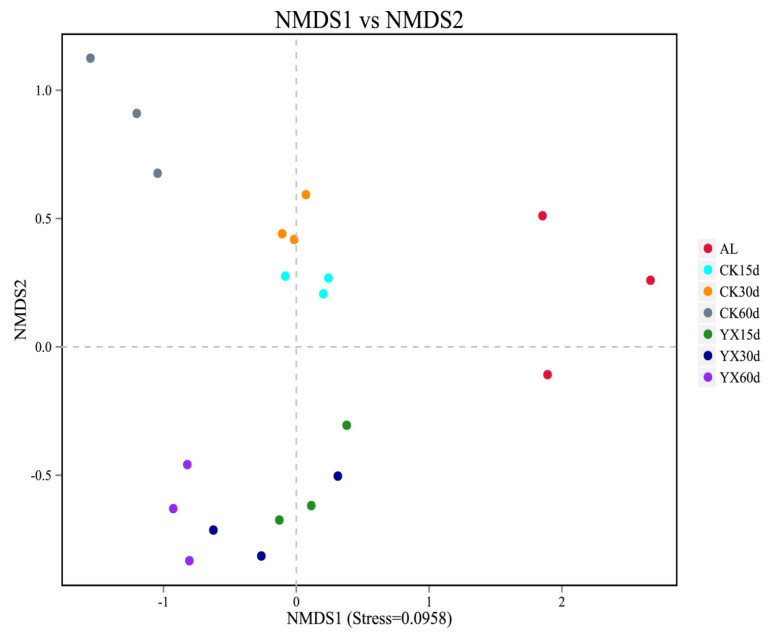
Non–metric multidimensional scaling (NMDS) plot based on OTU level in alfalfa silage. AL, alfalfa material before ensiling; CK, control; YX, inoculated with commercial LAB YX; the numbers following CK and YX stand for ensiled days of silage.

After ensiling, the variance of the bacterial community structure was demonstrated by NMDS. There was distinct separation between the fresh materials and treated silages (Figure 4). The clear separation between CK and YX silages indicated that the distribution of the bacterial community was shifted by LAB inoculations. The samples at 60 d could be separated from the samples at 15 and 30 d in CK silage. The distribution of the bacterial communities was similar among the three silage periods in YX silage.

**Figure 5:**
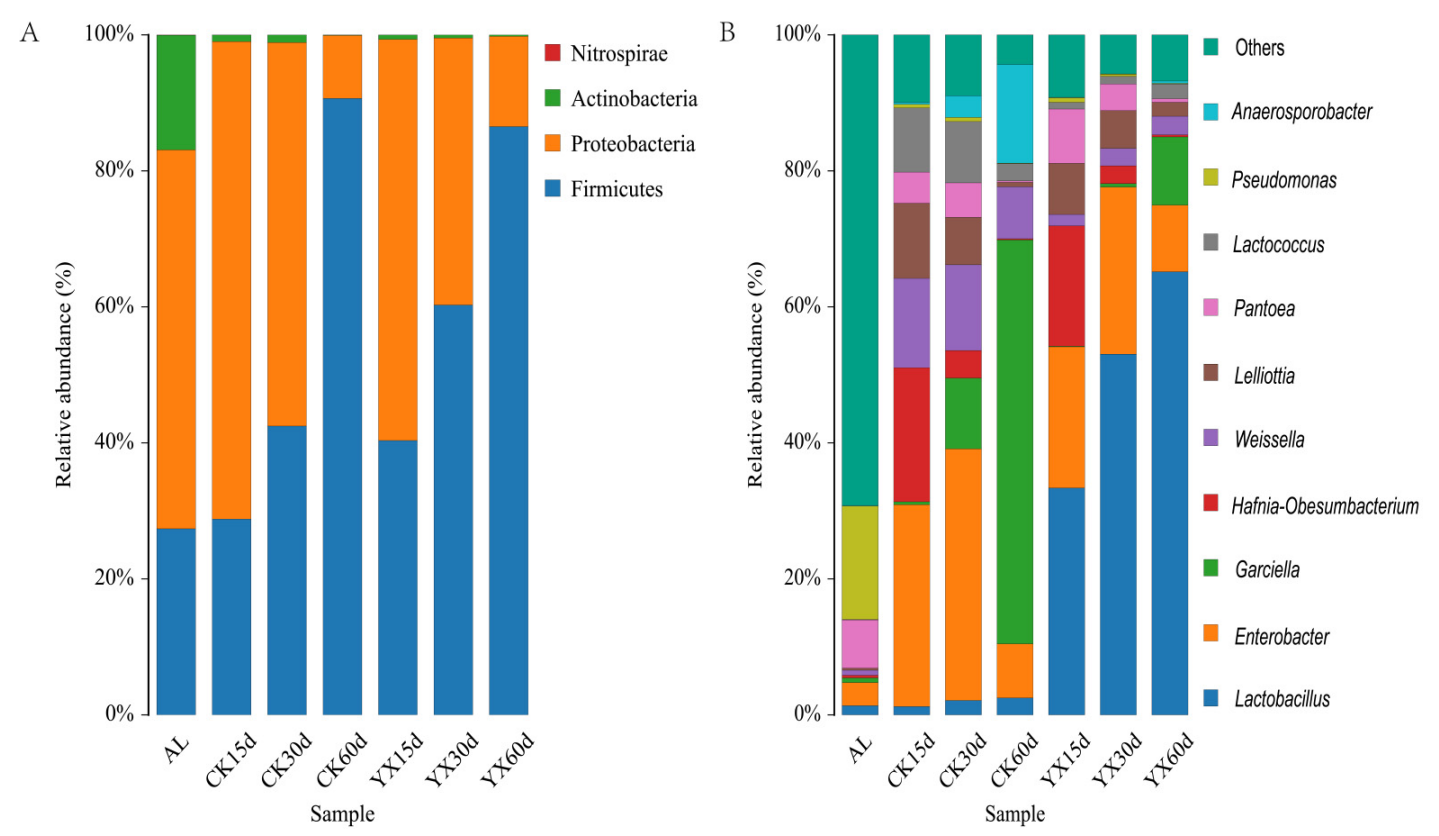
Bar plots of bacterial community and relative abundances by phylum (**A**) and genus (**B**) in alfalfa silage. AL, alfalfa material before ensiling; CK, control; YX, inoculated with commercial LAB YX; the numbers following CK and YX stand for ensiled days of silage.

The bacterial community structures of pre-ensiled and silage samples at the phylum and genus levels are shown in Figure 5. The phyla Proteobacteria (55.71%), Firmicutes (27.37%), and Actinobacteria (16.87%) were the main epiphytic bacteria in fresh materials (Figure 5A). In the CK silage, Proteobacteria and Firmicutes were the predominant phyla; with increased ensiling time, the relative abundance of Proteobacteria decreased, while that of Firmicutes increased. Compared with CK silage, the relative abundance of Firmicutes in YX silage was higher at 15 and 30 d, and no different at 60 d.

Genera *Enterobacter* (33.93%), *Pseudomonas* (16.67%), and *Pantoea* (7.09%) dominated the epiphytic bacterial community of alfalfa (Figure 5B). A low abundance of LAB species was exhibited in the alfalfa epiphytic bacterial community, including *Lactobacillus* (1.35%), *Weissella* (0.74%), and *Lactococcus* (0.07%). After ensiling, completely different microbial community dynamics were observed in CK and YX silages. In the CK silage, after 15 d of ensiling, the dominant microbial genera were *Enterobacter* (29.68%), followed by *Hafnia-Obesumbacterium* (19.71%), *Weissella* (13.14%), *Lelliottia* (11.05%), and *Lactococcus* (9.46%); *Enterobacter* (37.00%) peaked at 30 d and was lower at 60 d. With increasing time of ensiling, the relative abundances of *Hafnia-Obesumbacterium*, *Lelliottia*, *Weissella*, and *Lactococcus* declined, and *Garciella* and *Anaerosporobacter* increased; *Garciella* became the dominant genus at 60 d, with relative abundance of 59.26%. In the YX silage, *Lactobacillus* increased continuously during the ensiling process, and peaked (64.76%) at 60 d; proportions of *Enterobacter* and *Hafnia-Obesumbacterium* decreased from 15 to 60 d; the relative abundance of *Garciella* reached 9.88% at 60 d.

**Figure 6:**
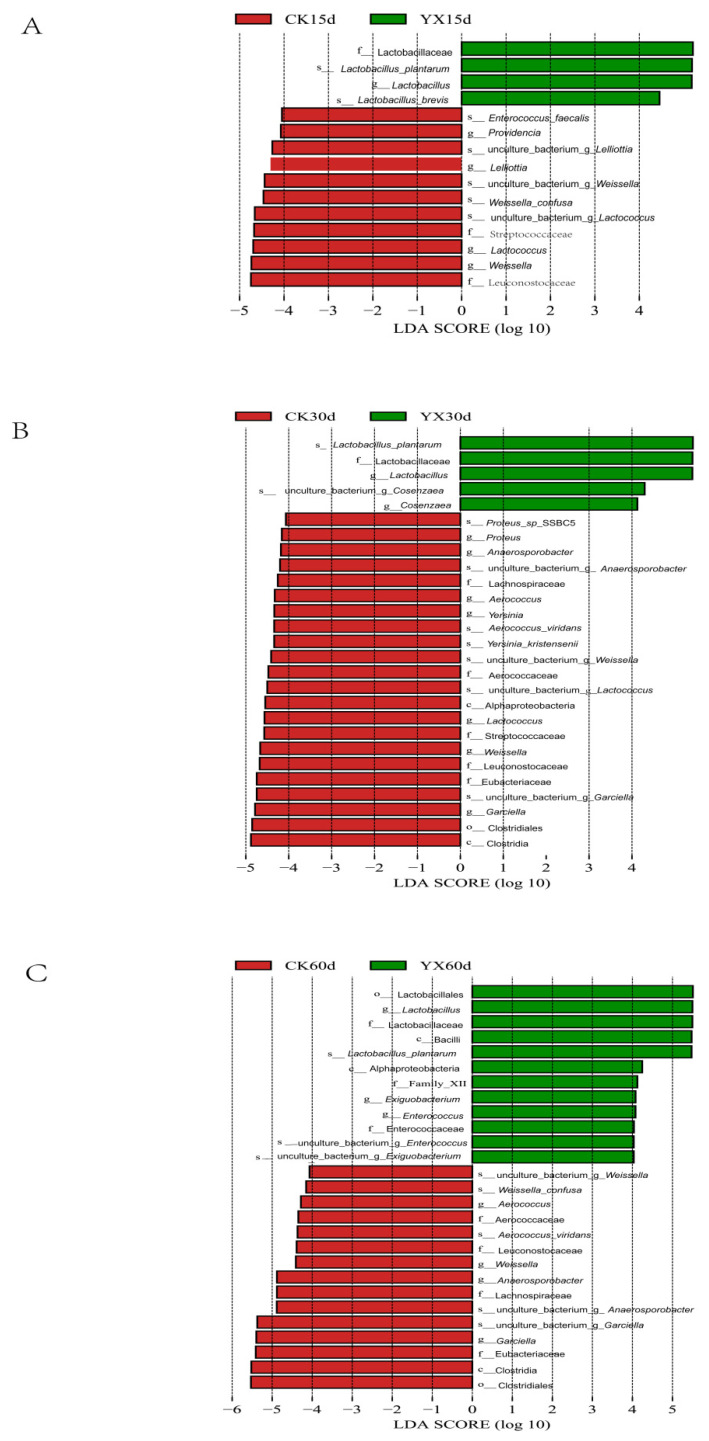
Comparison of microbial variations using LEfSe analysis for 15 d (**A**), 30d (**B**), and 60 d (**C**). CK, control; YX, inoculated with commercial LAB YX; the numbers following CK and YX stand for ensiled days of silage.

The LEfSe analysis was used to identify the taxa that most likely explained the differences in bacterial community structures between the CK and YX silages after 15, 30, and 60 d of ensiling (Figure 6). Compared with CK silage, YX silage had significantly higher abundance of *Lactobacillus* at each ensiling time and significantly higher abundance of *Enterococcus* at 60 d, but had significantly lower relative abundance of *Weissella* at each ensiling time, significantly lower abundance of *Lactococcus* and *Lelliottia* at 15 d, and significantly lower abundance of *Garciella* and *Anaerosporobacter* at 30 and 60 d (all *p* < 0.05).

### 3.3. Relationships between Fermentation Parameters and Bacterial Community

**Figure 7:**
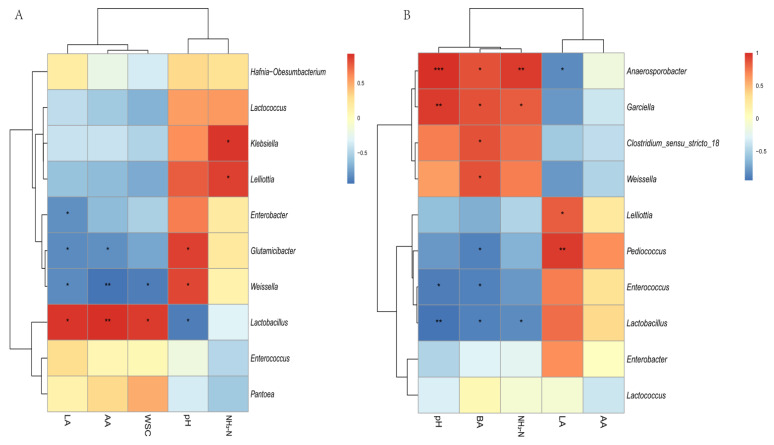
Spearman correlation heatmap of abundance of the top 10 abundant bacterial genera and fermentation properties in alfalfa silage for 15 d (**A**) and 60 d (**B**). LA, lactic acid; AA, acetic acid; BA, butyric acid; NH_3_-N, ammoniacal nitrogen; WSC, water-soluble carbohydrate. * *p* < 0.05; ** *p* < 0.01; *** *p* < 0.001.

Spearman’s correlations were used to study the relationships among the top 10 most abundant genera and fermentation parameters (Figure 7). At 15 d, pH was positively correlated with *Weissella* and *Glutamicibacter* and negatively correlated with *Lactobacillus* (*p* < 0.05); LA was positively correlated with *Lactobacillus* and negatively correlated with *Weissella*, *Glutamicibacter*, and *Enterobacter* (Figure 7A). There were positive correlations between NH_3_-N and two genera (*p* < 0.05), *Klebsiella* and *Lelliottia* (Figure 7A). At 60 d, pH was positively correlated with *Garciella* and *Anaerosporobacter* and negatively correlated with *Lactobacillus* and *Enterococcus* (*p* < 0.05); LA was positively correlated with *Lelliottia* and *Pediococcus* and negatively correlated with *Anaerosporobacter* (*p* < 0.05) (Figure 7B). NH_3_-N was positively correlated with *Garciella* and *Anaerosporobacter* and negatively correlated with *Lactobacillus*; BA was positively correlated with *Garciella*, *Anaerosporobacter*, *Clostridium_sensu_stricto_18*, and *Weissella* and negatively correlated with *Lactobacillus*, *Enterococcus*, and *Pediococcus* (*p* < 0.05) (Figure 7B).

## 4. Discussion

Generally, LAB is the main microbial inoculum affecting silage fermentation, because it can produce organic acids responsible for silage preservation; the LAB count must exceed 10^5^ cfu/g FM for optimum silage preservation [30]. In this study, fresh material showed chemical characteristics associated with poor silage quality, such as high BC, high moisture content, small numbers of epiphytic LAB, and low WSC content. These results showed that the alfalfa used was difficult to naturally ensile.

### 4.1. Changes in Fermentation Parameters, DM, and Structural Carbohydrates in Silages during Ensiling with or without LAB

Both pH and organic acids are important indicators to ensure silage quality. Previous studies reported that high moisture content was unfavorable for natural ensiling, leading to decreased organic acid concentration, increased pH, and a detrimental effect on the fermentation process for alfalfa silage [17,31]. In this study, the pH of CK silage increased rapidly to 7.07 at 60 d, which was related to insufficient LA fermentation and accumulation of NH_3_-N during the ensiling process. However, the pH of YX silage was significantly lower than that of CK silage at all silage stages, consistent with the results showing higher LA content of YX silage than of CK silage in all silage stages. The results showed that LAB as a silage additive accelerated the accumulation of LA and reduced the pH of the silage environment. Yang et al. also reported a similar effect of LAB as a silage additive, in which adding LAB in alfalfa silage accelerated the process of LA fermentation [18]. Although inoculation with LAB enhanced LA fermentation compared with CK silage, pH in the YX silage greatly exceeded the ideal level (<4.20) [32]. One possible reason for this was that the lack of WSC and high BC in alfalfa led to insufficient LA fermentation. In addition, for well-preserved silage, BA concentration should be <10 g/kg DM, with BA usually produced by Clostridia [33]. In this study, the BA concentration in CK silage was >10 g/kg DM at 60 d, indicating that the natural ensiling was unsatisfactory.

NH_3_-N is generally considered to be the result of amino acid deamination and decarboxylation, which reduce the nutritional value of silage in the ensiling process [34]. At 60 d, the CK silage became putrid, a pungent odor was released when silage bags were opened, and the texture was sticky and smooth; compared with CK silage at 30 d, the pH and NH_3_-N concentration were much higher, as was LA and WSC consumption, and BA was also detected. This is consistent with the typical characteristics of Clostridia fermentation. Kung et al. reported that the effects of plant and microorganism proteolytic enzymes may be typical causes of NH_3_-N accumulation [35]. The NH_3_-N concentration of YX silage at 15 and 30 d was not significantly (*p* < 0.05) lower compared with CK silage. One of the reasons may be that most plant protein hydrolases in alfalfa silage showed high activities at pH 5.0–6.0 [36], and the pH value of YX silage was in this range. In addition, another possible reason was that the inhibition of LAB of YX on proteolytic microorganisms was weak in these two silage stages. The DM was significantly (*p* < 0.05) lower in CK than in YX silage at 60 d, and may be because BA fermentation was greater in CK than in YX silage, resulting in greater DM loss of CK silage—a similar outcome was found by Liu [31]. In the present study, the structural carbohydrate degradation curve showed that LAB played a role in lignocellulose degradation to improve the fermentation quality of silage, which was similar to our previous research results [25]. A marked reduction in ADL and cellulose concentration occurred in the YX silage in all silage periods, probably because the higher organic acid accumulation in the YX silage promoted acid hydrolysis of the ADL and cellulose.

### 4.2. Changes in Bacterial Community in Silages during Ensiling with or without LAB

In this study, the diversity of the bacterial community of alfalfa silage was lower than that of fresh material, and may be due to the large increase in some bacteria with good adaptability to the conditions of ensiling. Similar trends were also reported by Zheng et al. [17]. The Shannon indexes were similar between the CK and YX silages at 60 d, and were lower in both silage groups than at 15 and 30 d. This may be related to the significant dominance of *Garciella* in CK silage and *Lactobacillus* in YX silage at 60 d.

The NMDS showed clear separation in bacterial community composition between fresh material and alfalfa silages (Figure 4). Ni et al. and Yang et al. also reported significant changes in microbial communities of fresh material and silage, and found that microbial communities of silage could not be predicted by those in fresh material because some species in fresh material were inhibited or inactivated during the ensiling process [18,37]. Divisions in our plots representing silage with or without inoculant indicated that LAB inoculation clearly changed the distribution of the bacterial community, consistent with differences in fermentation quality between the two silage groups [38].

Among many factors that can affect the fermentation process of silage, the dominant microbial species often determine the silage quality [39]. Therefore, analyzing the changes in fermentation parameters and microbial composition during ensiling is helpful in understanding the silage process and for improving silage quality [40]. In this study, Proteobacteria were the most abundant bacteria in the pre-ensiled samples and 15 d silage. Proteobacteria play a significant role in organic matter degradation and carbon and nitrogen cycling during anaerobic digestion [41]. However, Firmicutes replaced Proteobacteria as the dominant phylum at 60 d. Bao et al. reported a similar result for silage of the legume *Medicago* [42]. Keshri et al. concluded that low pH or anaerobic conditions during ensiling favored the growth of species of Firmicutes, similar to our study in which Firmicutes was prevalent after 60 d of ensiling [43].

During ensiling, LAB play an important role in silage fermentation. In this study, fresh alfalfa had a high moisture content and low relative abundance of epiphytic LAB. Therefore, adding LAB was an effective way to improve quality of alfalfa silage. Different species and characteristics of LAB may affect the fermentation process [44]. In this study, the major LAB after ensiling were *Lactobacillus*, *Lactococcus*, and *Weissella*; among these, *Lactobacillus* plays a key role in silage fermentation. It can produce LA, reduce pH, reduce the relative abundance of undesirable bacteria, and often becomes a dominant genus in various high-quality silages [45,46]. In this study, the relative abundance of *Lactobacillus* in the natural silage group was very low during the whole silage period, and adding LAB of YX significantly (*p* < 0.05) increased the *Lactobacillus* abundance with longer silage time, consistent with the higher LA concentration and lower pH in YX than in CK silage, indicating that adding LAB of YX had a positive effect on fermentation quality. We speculate that the increased *Lactobacillus* is likely to be the LAB YX strain, but unfortunately, this study cannot accurately determine the proportion of LAB YX in *Lactobacillus*, because NGS, which was used in our study, can only classify and identify microorganisms to genus level [47]. *Lactococcus* and *Weissella* are cocci-shaped LAB, and can initiate LA fermentation in the early stage of ensiling [30]. After the onset of ensiling, the main LAB in the CK silage were *Lactococcus* and *Weissella*, and their relative abundances were much higher than those in the YX silage. This is possibly because *Lactococcus* and *Weissella* were early colonizers, and could grow faster under ensiling conditions. Yang also reported that *Lactococcus* and *Weissella* started LA fermentation and grew vigorously during the early stage of ensiling in untreated silage [48]. However, *Lactococcus* and *Weissella* in YX silage could be outcompeted by *Lactobacillus* due to adding LAB of YX. Yang et al. made a similar report in which *Lactobacillus* outnumbered all other genera after inoculation with *Lactobacillus plantarum* [18]. Enterobacteriaceae are generally considered to be undesirable during silage because they can ferment LA into AA and other products, resulting in nutritional loss [37]. Enterobacteriaceae can survive in low pH environments and compete with LAB for WSC [49]. In our study, the relative abundance of Enterobacteriaceae, including *Enterobacter*, *Hafnia-Obesumbacterium*, and *Lelliottia* exceeded 38.8% in both silage groups at 15 and 30 d. Compared with CK silage, adding LAB of YX did not significantly reduce the abundance of *Enterobacter* and *Hafnia-Obesumbacterium*, possibly due to a large number of Enterobacteriaceae on the surface of fresh alfalfa. Therefore, in practical application, it is better to consider the dose–response and/or the inter-relationship between indigenous bacteria and the LAB YX added. Additionally, this was also related to the limitation of acid production ability of LAB of YX under conditions of high moisture content and low WSC; the weak inhibitory effect of LAB of YX on Enterobacteriaceae may also be another reason. Therefore, despite adding LAB of YX, *Lactobacillus* could not quickly become absolutely dominant in the community. Similar results were reported by Guo et al. in which *Enterobacter* was one of the two dominant bacteria after ensiling, especially in LAB-treated silage [50]. In our study, the improvement effect of LAB of YX on the silage quality of high-moisture alfalfa was limited. Kung also reported that compared with untreated silage, homofermentative LAB may reduce the pH of silage, but the degree of reduction may be insufficient to prevent undesirable bacteria in alfalfa growth, depending on the specific situation [31,51]. Pahlow et al. considered that when LA fermentation was inadequate to rapidly decrease the pH of a silage system, Clostridia fermentation easily occurred [52]. *Garciella* is an anaerobic and thermophilic bacterial genus of the class Clostridia, and is undesirable in silage because it can lead to excessive protein degradation, DM loss, and BA production [53]. *Garciella* can grow in the pH range of 5.5–9.0, and its optimum pH is 7.5. However, growth of Clostridia can be completely inhibited only for pH < 4.2 [33]. Clostridia are known to be particularly sensitive to water availability, and they require wet conditions for proliferation [48]. In this study, the CK silage showed severe corruption and *Garciella* became the dominant genus at 60 d, possibly due to the high moisture concentrations. Similarly, Zhang et al. found that with longer ensiling time, *Garciella* played a more important role in Clostridia fermentation, and that the presence of *Garciella* led to alfalfa being more difficult to ensile [17]. Therefore, it can be further speculated that *Garciella* is one of the dominant bacteria in Clostridia fermentation. *Anaerosporobacter* is similar to *Garciella*, and also belongs to class Clostridia [54]. It is speculated that *Anaerosporobacter* may play the same role as *Garciella* in silage. Compared with CK silage, the abundance of *Garciella* and *Anaerosporobacter* was significantly (*p* < 0.05) lower after adding LAB of YX, consistent with the lower pH and NH_3_-N concentration and absence of BA in YX silage at 60 d.

### 4.3. Relationships between Fermentation Parameters and Bacterial Community

A Spearman’s correlation heatmap (Figure 7) was used to elucidate relationships among the fermentation parameters and the microbial community. It has been reported that the plant proteases produced by plant respiration at the beginning of ensiling may be related to the synthesis of NH_3_-N. With longer ensiling time, the synthesis of NH_3_-N is also related to the activity of protease produced by some microorganisms [55,56]. In this study, the bacteria associated with NH_3_-N differed for different periods. The NH_3_-N concentrations were significantly (*p* < 0.05) positively correlated with *Lelliottia* and *Klebsiella* at 15 d (both Enterobacteriaceae). It was reported that proteases produced by Enterobacteriaceae were related to synthesis of NH_3_-N [57]. Compared with YX silage, the concentration of NH_3_-N of CK silage was significantly (*p* < 0.05) higher at 60 d, possibly because *Garciella* became the dominant bacteria in CK silage at that time, consistent with the significant (*p* < 0.05) positive correlation between *Garciella* and NH_3_-N at 60 d. The appearance of Clostridia was undesirable because they can use protein and WSC to produce BA and consequently affect silage quality due to the unpleasant odor of BA [17]. A large amount of BA was detected in CK silage at 60 d, consistent with the significant (*p* < 0.05) positive correlation of *Garciella* and *Clostridium_sensu_stricto_18* with BA. *Anaerosporobacter* was also significantly (*p* < 0.05) positively correlated with NH_3_-N and BA, and may have similar effects to *Garciella* during ensiling. *Weissella* was significantly (*p* < 0.05) positively correlated with BA at 60 d, likely because BA was only detected at 60 d in CK silage and the relative abundance of *Weissella* was higher in CK than in YX silage. It was reported that the ability of *Weissella* for LA fermentation was lower than that of *Lactobacillus* [30], so CK silage with a higher pH than YX silage failed to inhibit undesirable microbial activity. *Lactobacillus* was positively correlated with LA concentration and negatively correlated with pH, consistent with the general recognition that *Lactobacillus* is the main producer of LA and plays an important role in reducing pH during ensiling [30,46,58].

The experimental results indicate that it is important to develop high-efficiency LAB additives with strong acid production ability and the ability to effectively inhibit the growth of Enterobacteriaceae, so as to improve the quality of alfalfa silage.

## 5. Conclusions

In conclusion, naturally ensiled alfalfa with high moisture content was prone to rot, and the relative abundance of *Enterobacter*, *Hafnia-Obesumbacterium*, *Garciella*, and *Anaerosporobacter* showed these to be the dominant genera. Adding LAB of YX delayed the rot process to a certain extent by lowering pH, promoting LA and AA accumulation, inhibiting BA formation, increasing the abundance of *Lactobacillus*, and decreasing the abundance of *Garciella* and *Anaerosporobacter*. However, the quality of silage with LAB of YX addition was still unsatisfactory, because the pH in silage was not reduced to a satisfactory value, and the growth of *Enterobacter* and *Hafnia-Obesumbacterium* could not be effectively inhibited. Thus, it is a focus to develop high-efficiency LAB additives with strong acid production ability and effective inhibition of the growth of Enterobacteriaceae in a future study.

## Data Availability

Data are contained within the article.
